# On the net primary productivity over the Arabian Sea due to the reduction in mineral dust deposition

**DOI:** 10.1038/s41598-022-11231-7

**Published:** 2022-05-11

**Authors:** Chakradhar Rao Tandule, Mukunda M. Gogoi, Rama Gopal Kotalo, S. Suresh Babu

**Affiliations:** 1 Aerosol and Atmospheric Research Laboratory, Department of Physics, Sri Krishnadevaraya University, Anantapur, 515003 India; 2 Space Physics Laboratory, Vikram Sarabhai Space Centre, ISRO, Thiruvananthapuram, 695022 India

**Keywords:** Atmospheric science, Environmental impact

## Abstract

The dust plume tracks from the Middle East and Eastern Africa to the Indian subcontinent have an impact on the atmospheric and ocean biogeochemistry of the Arabian Sea (AS). Here, we present the impact of dust on net primary productivity (NPP) over the AS using satellite-based observation and model simulation. Seasonal episodes and long-term trends in dust optical depth (DOD), dust mass flux (DMF) and dust deposition flux (DDF) from 2007 to 2020 are quantified. Nearly 32% of the total dust is advected to the AS during transport (maximum in JJA; DMF ~ 33.1 Tg year^−1^ ~ 56% of annual and DDF ~ 5.5 Tg year^−1^ ~ 63% of annual). Over the last one and half decades, there has been a statistically significant decreasing trend in DOD, associated with precipitation, enhanced vegetation index and surface soil moisture over the landmasses in the proximity of the AS. Similarly, the depletion in DDF suppresses the NPP over different regions of the AS, especially over the central AS, where the oceanic supply of nutrients is limited.

## Introduction

In the complex mixture of aerosols in the earth's atmosphere, mineral dust of natural origin is most abundant by mass in the troposphere next to the sea salt aerosols^1^. Having an average lifetime of ~ 2.8 days^2^ in the atmosphere, mineral dust plays a crucial role in air quality^3^, human health^4^, terrestrial and oceanic biogeochemical cycle^5^, cloud lifecycles and monsoon precipitation^6–8^, and radiative forcing^9^ in the near and far off locations. On a global scenario, the Middle East and North Africa regions are the most active natural dust source regions^10^, contributing up to 80% of the global dust burden^2^. Western Africa, Horn of Africa, Arabian Peninsula, eastern Kenya, and Iran–Afghanistan–Pakistan (IAP) are dominant dust emitting sectors during March–April–May (MAM) and June–July–August (JJA), affecting the global aerosol burden and radiation budget^10^. In order to understand the major transport pathways and the amount of dust transported from the dust source regions to distant locations, their altitudinal spread and deposition rates during transport, a comprehensive study using satellite observations from space-borne platforms is ideal for quantifying the seasonal signatures, inter-annual variations and impact of dust on surface biogeochemistry. Kaufman et al*.*^11^ introduced the quantitative estimation of dust from the satellite observations, following subsequent development by other investigators^12,13^

Dust is considered one of the major sources of nutrients to the biogeochemistry over the ocean^5,14^. Thus, perturbations in dust deposition over the ocean can have a notable impact on net primary productivity (NPP), as the growth of NPP over any region is related to the supply of macro and micronutrients, driven by the upwelling of nutrient-rich water from deep oceans towards the ocean surface^15^, sea surface temperature^16^, deposition of dust^14^, light intensity^17^, eddies, winter convection^18^, the wind stirring, ocean currents^19^, river input^20^, etc. In this study, we focus on the Arabian Sea (AS, the north-western part of the Indian Ocean) because of its proximity to the arid and semi-arid regions of the Middle East and Eastern Africa (MEEA). The dust emitted from the MEEA is often transported eastwards across the AS, making it the second most dust-laden oceanic region (especially the western and northern parts of the AS) among the global oceans^9^. There is an enormous contribution of dust from Central Asia, followed by the southern Arabian Peninsula, Central Africa, East Africa, and West Africa to the north Indian Ocean^21^. As the southwest monsoon associated with the low-level Findlater jet is a conduit for natural aerosols (e.g., dust and sea salt) influencing monsoon precipitation on short timescales^6^, several studies have focused on the qualitative analysis of dust^22,23^ and its impact on the radiation budget^9^ and monsoon system over India^7^. However, quantitative analysis of dust aerosols over the AS is very limited except for a few studies reporting some episodic events^24,25^ and several others focussing on the anthropogenic impact of aerosols over the AS^26,27^.

The AS is also considered as one of the most biologically productive oceanic regions in the globe^25^. The primary production in the oceanic regions account for roughly half of global biospheric production due to the availability of nutrients and is vital to most marine ecosystems^28^. As far as climate is concerned, increasing dust deposition into the oceans may result in an upsurge of productivity and thus a decrease in CO_2_ in the atmosphere and vice-versa^29^. Thus, it is critical to assess whether net primary productivity (NPP) is changing (on a long-term scale) and, if so, how rapidly and what factors are influencing those particular changes? Most investigations have focused on the supply of nutrients via dust deposition using model simulations^14,30,31^ and a very few episodically^25,32,33^ or based on campaign mode studies^34,35^. Thus, there is a lag in the long-term quantitative analysis (observational) of dust deposition over the oceanic regions, especially over the AS and its impact on oceanic biogeochemical cycles.

In the above backdrop, we quantify the dust transport and deposition flux into the AS based on clear sky Cloud-Aerosol Lidar and Infrared Pathfinder Satellite Observation (CALIPSO) data during 2007–2020. The domain of interest (0°N to 40°N, 30°E to 76°E) is shown in the supplementary Fig. S1 online. Further, the impact of dust deposition on NPP over the study domain is investigated. The monthly NPP is obtained from Moderate Resolution Imaging Spectroradiometer (MODIS) Chlorophyll based Vertically Generalized Production Model (VGPM)^36^ simulations (Oregon State University, Ocean Productivity website). Since the development of NPP is not due to dust alone, the role of nitrate, ocean mixed layer depth and sea surface temperature (SST) are also examined. The monthly nitrate and ocean mixed layer depth data were obtained from the NASA Ocean Biogeochemical Model (NOBM).

## Results and discussion

### Dust mass and deposition flux

**Figure 1:**
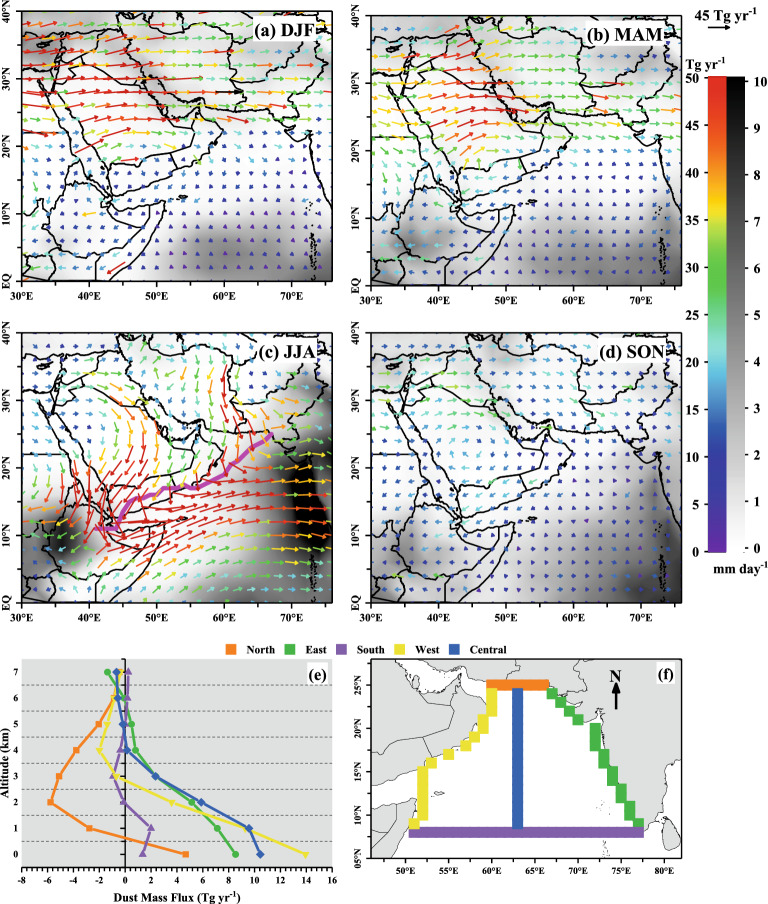
From 2007 to 2020 (**a–d**), seasonal and spatial distribution of dust mass flux (DMF) in terms of magnitude and direction, with color scale and the length of the vector showing the magnitude of DMF. The seasonal precipitation rate, derived from the Global Precipitation Measurement (GPM), is also shown (grey contour). (**e**) The average zonal and meridional DMF at the boundaries of the AS (as shown in the bottom right panel—(**f**) at different vertical regimes during JJA. The values of DMF are positive (negative) for north (south) boundaries when the direction of flux is from south to north (from north to south). For the west, central and east boundaries, positive (negative) DMF represents the direction of flux from west to east (from east to west). The location of ITD (the area of near-zero meridional wind at 850 hPa estimated from the MERRA-2 reanalysis data for the period 2007–2020) is shown in (**c**) by the solid magenta line.

Based on the long-term (2007–2020) estimates of dust mass flux (DMF; Fig. 1a–d), we find that the AS experiences the highest dust incursion during JJA (33.1 ± 12.6 Tg year^−1^), which is about 56% of the annual DMF. DMF is below 20% during other seasons and lowest in September–October–November (SON) (~ 4.53 Tg year^−1^). The dominant source regions of dust in JJA are north–east Africa, the Arabian Peninsula and IAP, located southwest, west and north of the AS. Dust from these regions is transported and accumulated over the AS by the confluence of Red Sea winds, the Shamal winds over the Arabian Peninsula, and the low-level Findlater Jet of the southwest monsoon through Somalia. It is further aided by the Inter-Tropical Discontinuity (ITD) (Fig. 1c) over the southeast Arabian coast, coalescing the dusty air masses from the Arabian Peninsula and forming the dust-laden area over the western AS^37^. Jin et al*.*^7^ hypothesized that dust emissions over the Arabian Peninsula could be enhanced due to the topographic heating over the Iranian Plateau, thus strengthening the Shamal winds and the Indian summer monsoon (ISM) circulations. Even though westerly winds blow in gusts over the northern AS, dust build-up is not substantial during the rest of the seasons.

To understand the vertical extent of dust entering/leaving the AS region during JJA (principal dusty season over the AS), the mean (climatological) zonal DMF at the west, central and east boundaries, and meridional DMF at the north and south boundaries of the AS at different altitudinal sectors (between surface and 7 km altitude) are examined (Fig. 1e,f). As dust beyond 7 km is negligible, this might cause a minor impact on oceanic biogeochemistry through its deposition over the AS. A few earlier studies have also shown that dust extinction beyond 7 km is very low/negligible over the AS (e.g., Kuhlmann and Quaas^38^; Song et al*.*^39^). Thus, in the following sections, only the altitudinal sectors from surface to 7 km are considered for discussion. It is observed that dust enters the AS region primarily through the west (26.8 Tg year^−1^) and northern (20.8 Tg year^−1^) boundaries. The dust influx from the other two boundaries is negligible (< 4 Tg year^−1^). During the transport, about 32% of dust is advected to the AS from the dust plume track spanning from the Middle East to the Indian subcontinent. This also indicates that a significant amount of dust is transported to the Indian mainland. However, precipitation during JJA plays a crucial role in reducing the extensive transport of dust to the far-off locations of the Indian peninsula. There occurs efficient scavenging and wet deposition of dust over the western Ghats of India associated with the intense precipitation during JJA^8^.

The vertical extent of DMF is mostly confined below 3 km altitude while flowing across the west, east and south boundaries of the AS. This could be attributed to the strong low-level Findlater jet associated with the southwest monsoon. The zonal DMF decreases from west to east, passing through central boundaries at the surface to 2 km altitudes. Interestingly, the vertical span of westerly DMF increases (0–5 km) during its exit at the eastern side of the AS, while the DMF at the western AS lies within 2 km altitude. These features are very well synchronised with the convective processes observed during ISM (longitude-altitude cross-section of DMF averaged from 10°N to 20°N latitudes over the AS during JJA, see Supplementary Fig. S2 online). A remarkable in-flux of dust from the IAP region distributed vertically within the altitude range of 2–7 km is also observed in the northern boundary. The greater vertical extent of meridional DMF in JJA (as high as 6 Tg year^−1^ at 2 km altitude) is due to the combination (ITD) of dusty air masses from low-level Findlater jet and the Levar winds (see Supplementary Fig. S3c online).

**Figure 2:**
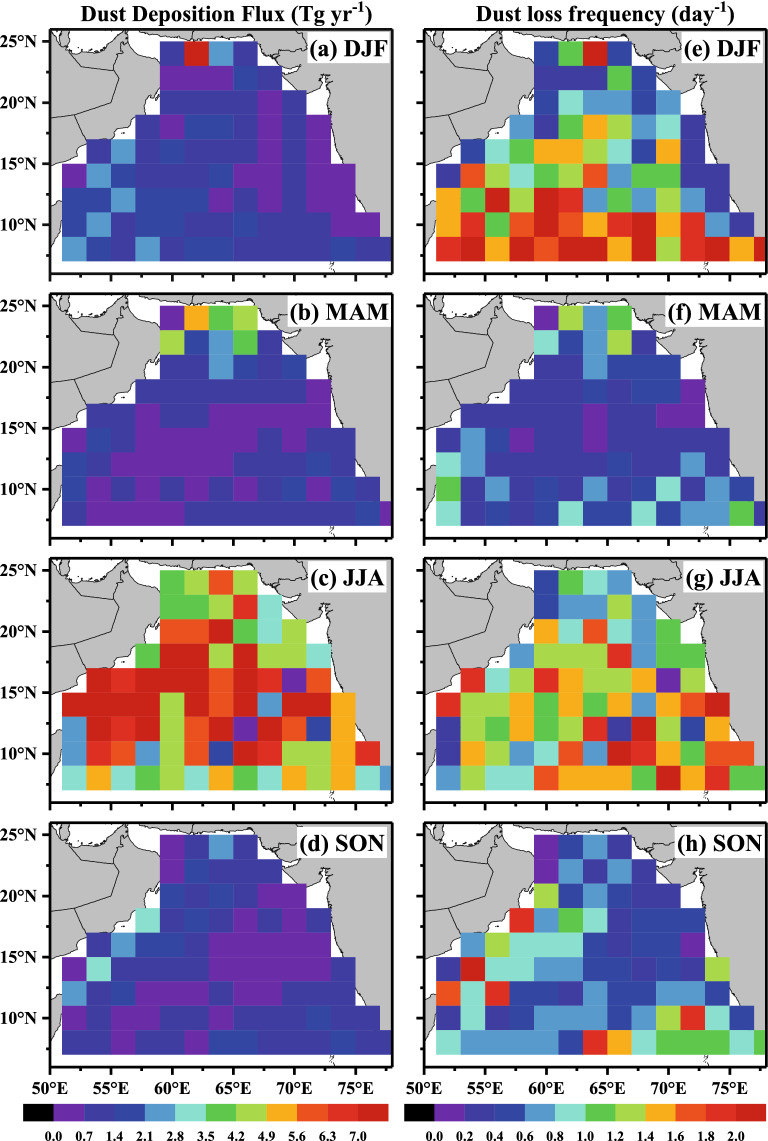
Spatial distribution (mean picture during 2007–2020) of dust deposition flux (**a–d**) and dust loss frequency (**e–h**) at different seasons over the Arabian Sea.

Considering the divergence of zonal and meridional DMF, dust deposition flux (DDF) into the AS is derived, and its spatiotemporal variability is examined (Fig. 2a–d). Associated with high DMF, DDF over the AS is highest in JJA (mean 5.6 ± 2.6 Tg year^−1^), with maximum dust deposition of 26.8 Tg year^−1^. Over the northern AS (20°N–25°N), the low-level northward winds (see Supplementary Fig. S3c online) lift the dust particles from arid soils and create a low-level dusty environment along with the subsidence of the vertical winds, which produces a large amount of dust deposition during JJA (4.8 Tg year^−1^). Over the southeastern part of the AS (between 08°N and 14°N), similar phenomena are observed during JJA associated with extensive precipitation over this region (Fig. 1). Relatively high DDF appears in the central AS (14°N–19°N), over which the intensity of precipitation is also high (Fig. 1c). In general, DDF is higher near the coast of the southeast Arabia Peninsula and generally decreases away from the coast. Overall, the spatiotemporal variability in retrieved DDF is consistent with atmospheric circulations and precipitation patterns. The spatial patterns of DDF observed in this study are also consistent with the modelled results (during 1997–2004) reported by Patra et al*.*^30^*.*

In terms of dust loss frequency (DLF, Fig. 2e–h), large differences in seasonal and spatial patterns are seen from that between DMF (Fig. 1a–d) and DDF (Fig. 2a–d). The subsidence of the vertical winds leads to the high DLF over northern and western parts of the AS, where no/little precipitation is observed. On the other hand, DLF over the southern and eastern parts of the AS (far away from the potential dust source regions) shows very good associations with moderate to high precipitation at different seasons. By definition, the reciprocal of DLF represents the mass-weighted dust lifetime (DLT) in the atmosphere^40^. The seasonal mean value of DLT is lowest in JJA (0.4 ± 0.2 days) while varying between 1.6 ± 0.3 days in MAM, 1.0 ± 0.3 days in SON and 0.6 ± 0.3 days in December-February (DJF). These observations suggest that the dust transported to the AS during JJA possesses a very short lifetime, either influenced by the intense scavenging process or blown away from the AS by the strong southwest monsoonal winds.

### Long-term trends

Several recent studies have reported a significant reduction in mineral dust loading in the atmosphere over the MEEA regions. For instance, Song et al*.*^39^ have reported the decreasing trend in dust optical depth (DOD) over the Middle East (−0.0038 year^−1^ *p* < 0.05) and the AS (−0.0013 year^−1^ *p* > 0.1) during 2007–2019. Another study by Lakshmi et al*.*^41^ have reported a significant decreasing trend in dust fraction and DOD over the northeast Indian Ocean (Bay of Bengal). Jin et al*.*^42^ have reported strong interdecadal dust variability over the AS between 2000 and 2016. The increase in precipitation leads to the decrease of the dust loading in the atmosphere by 10–20% over these regions, decreasing the direct surface radiative forcing^43^. Pandey et al*.*^44^ have attributed an increase in the pre-monsoon (MAM) rainfall over the Pakistan and Thar Desert (western part of India) region to the decreasing trend in DOD over South Asia.

**Figure 3:**
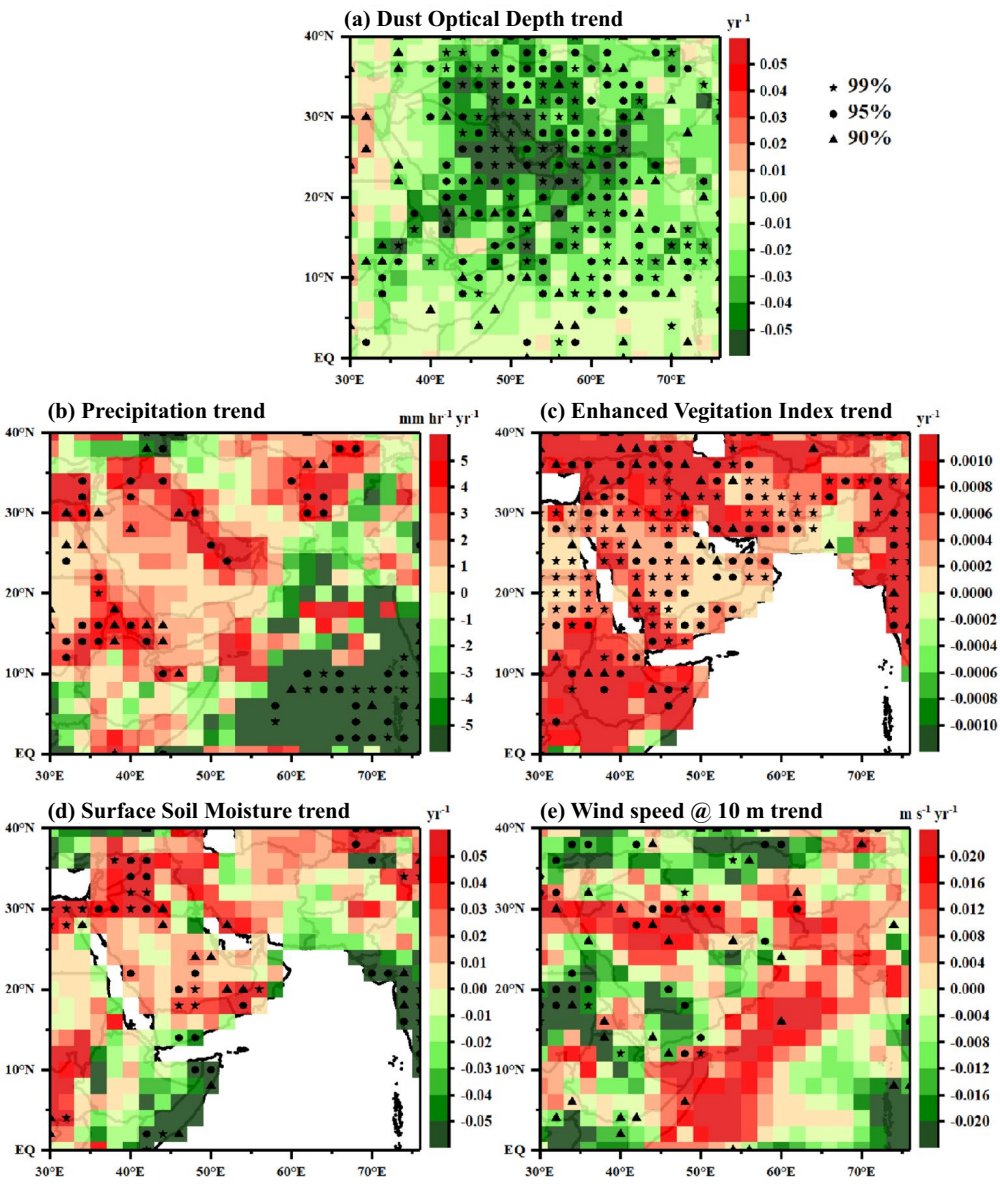
The annual trends in **(a)** dust optical depth (DOD from CALIPSO), **(b)** precipitation (PPT; from GPM), **(c)** enhanced vegetation index (EVI; from Aqua-MODIS C6), **(d)** surface soil moisture (SSM; from MERRA-2), and **(e)** wind speed (WS_10_; from MERRA-2) in JJA during 2007 to 2020 at each 2° × 2° cell over the study region. The trend is estimated using the linear regression, and the statistical significance is ensured with the Mann–Kendall test, and trends with a confident interval of 99% are shown as star, 95% as filled circle and 90% as filled triangle.

To have a deeper insight into the decreasing trends of dust over the AS, a comprehensive analysis (Fig. 3) of various parameters [viz., DOD, precipitation (PPT), enhanced vegetation index (EVI), surface soil moisture (SSM), and wind speed at 10 m above the surface (WS_10_)] which influence the production and transport of dust to the AS is carried out on an annual basis. The linear regression technique is applied for each 2° × 2° gridded data having the mean values for each year ranging from 2007 to 2020 and estimated the slope (termed as a trend), considering 2007 as the base year. The significance of the trend is tested using the Mann–Kendall test. As shown in Fig. 3, the decreasing trend in DOD over the potential dust source regions is statistically significant (Fig. 3a). DOD has decreased significantly across the western AS (most dust-laden region over the AS), decreasing at the rate of 0.02 year^−1^. Similarly, EVI (Fig. 3c) and SSM (Fig. 3d) over MEEA regions show a significant increasing trend along with increasing trends of precipitation over this region (Fig. 3b). The increasing trends in these parameters over the MEEA could reduce dust build-up and, hence, decrease in the DOD, despite the increasing trends in surface winds over the Arabian Peninsula region (Fig. 3e). As the precipitation over the red sea shows a statistically significant increasing trend, this can lead to wash out of most of the dust aerosols reaching the western part of the AS.

Thus, it can be summarized that increasing trends in precipitation, along with EVI and SSM, over the MEEA region are the major influencing factors towards the decreasing trends of dust loading over the AS. Pandey et al.^44^ and Jin and Wang^45^ also reported that the increased precipitation over the north-western Indian subcontinent increases EVI and SSM with decreased dust loading.

Over the dust source region, intense precipitation leads to an increase in wet removal of aerosols from the atmosphere, altering emission intensity due to SSM changes and vegetation growth. The vegetation is one of the critical parameters that influence the generation of dust aerosols. When the surface is covered with vegetation, it protects the underlying soils from lift-off due to surface winds and hence plays a vital role in modifying the quantity of dust emitted from that vegetated soil. There exists a negative relationship between increasing vegetation cover and the occurrence of dust storms, as stated in the IPCC 2019 special report with high confidence^46^. Also, changes in the groundwater/SSM can affect the vegetation, thereby generating atmospheric dust^47^. Hence, parameters like EVI and SSM significantly influence the observed changes in the dust loading over the study domain in connection with precipitation.

### Impact of dust on net primary productivity

**Figure 4:**
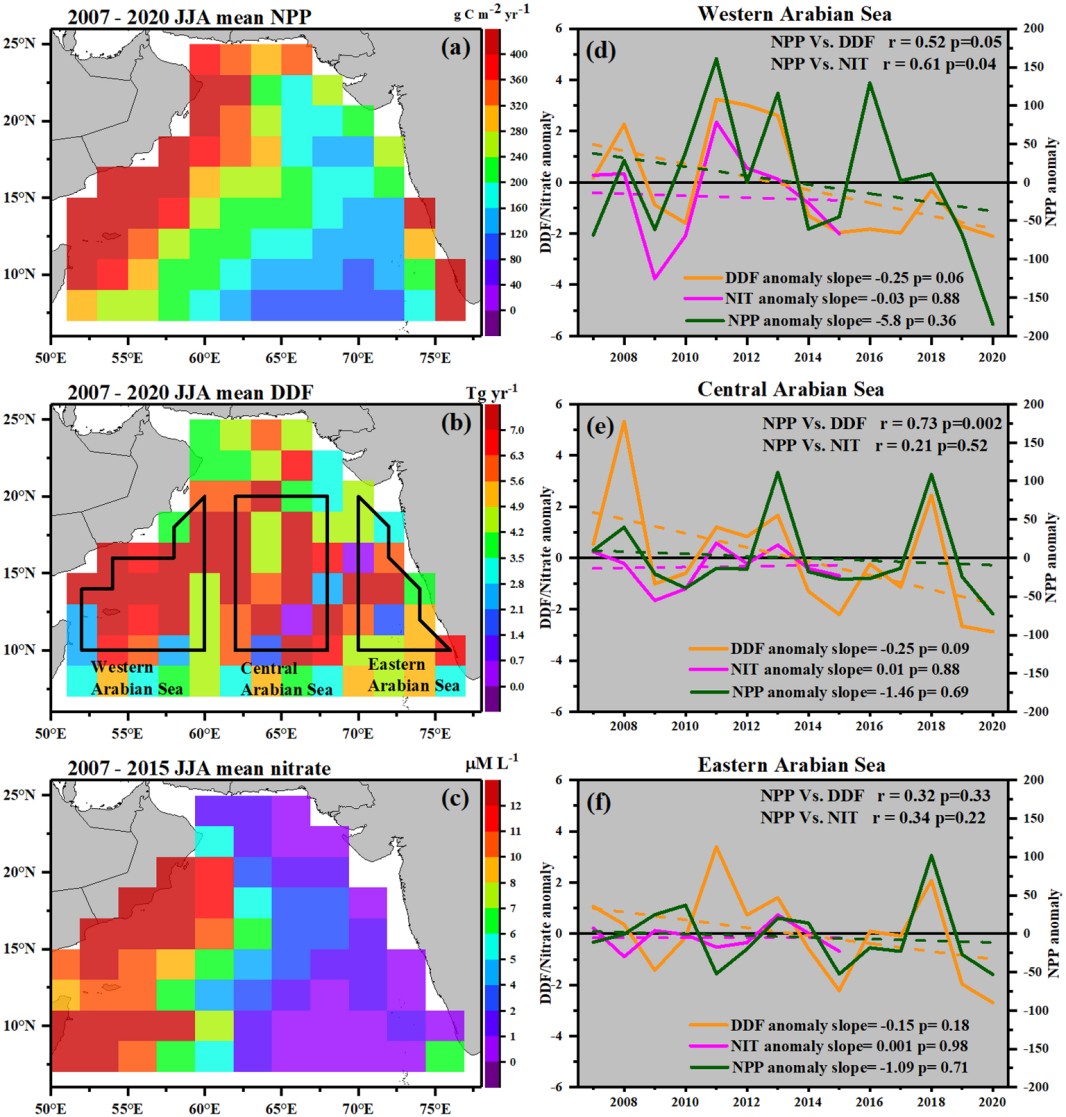
Spatial distribution of mean **(a)** NPP, **(b)** DDF, and **(c)** nitrate (NIT) during JJA. The geographical separation of the Arabian Sea region is based on dust deposition and is shown in **(b)**. The interannual trends of the NPP, DDF and NIT anomalies over the **(d)** western Arabian Sea, **(e)** central Arabian Sea and **(f)** eastern Arabian Sea. The interannual trends are estimated using linear regression, and their significance is tested using the Mann-Kendell test, and those statistics are shown at the bottom of each plot (**d–f**). The dependency of NPP on the DDF and NIT is shown using the correlation analysis. The Pearson's r values and *p*-values of each relating variable are shown on top of each plot (**d–f**). Note that the availability of NIT data from NOBM is only till 2015; the spatial distribution of mean NIT during JJA is calculated from 2007 to 2015.

To examine the impact of dust on NPP over the AS, the spatial distribution of NPP (2007–2020), DDF (2007–2020) and nitrate (NIT; 2007–2015) during JJA are examined. Considering the extensive dust deposition in the latitudinal band from 10°N to 20°N, this region is classified further (Fig. 4b) into the western Arabian Sea (WAS), central Arabian Sea (CAS), and eastern Arabian Sea (EAS), over which the inter-annual trends of NPP, DDF and NIT anomalies during JJA are analysed. The linear regression technique is applied for estimating the trend, and the statistical significance of the trend is ensured with the Mann–Kendall test. Also, to find out the dependency of NPP on DDF and NIT, Pearson's correlation method is applied to the interannual anomalies. Though the correlation analysis is not adequate to explain the variations and association of NPP with DDF, the long-term trends suggest that the perturbations in dust deposition can have a notable impact on NPP.

As seen from Fig. 4a, NPP rates are relatively higher over the western AS, the region where nitrate availability (Fig. 4c) and accumulation of dust are higher (Fig. 4b). This is also seen in the regional average (climatological) values of NPP, DDF and NIT during JJA (as given in Table 1), which decrease from the WAS to EAS. Interestingly, the decrease in NPP and NIT is steeper when compared to DDF. For example, the reduction in NPP (NIT) from WAS to EAS is ~ 57% (88%), whereas, for DDF, it is much lower (~ 31%).

**Table 1:** Regional average values of NPP (2007–2018), DDF (2007–2018) and NIT (2007–2015) during JJA.

	Western AS	Central AS	Eastern AS
NPP (g C m^−2^ year^−1^)	479.87 ± 89.16	208.18 ± 50.66	205.74 ± 52.96
DDF (Tg year^−1^)	7.12 ± 2.06	6.08 ± 2.23	4.89 ± 1.65
NIT (μM L^−1^)	12.32 ± 1.90	3.97 ± 0.98	1.47 ± 0.57

The higher concentration of NIT (Fig. 4c) over the WAS arises due to the coastal upwelling during JJA. As cold water from deeper layers lifts up to the ocean surface during upwelling, causes lowering of SST. Hence, SST can be an indicator of the upwelling over the AS. Kumar et al*.*^19^ have also reported that upwelling has the most significant impact on SST along the coastal regions of AS during JJA. As the spatial pattern of NIT resembles the SST distribution (see Supplementary Fig. S4b online), it signifies that ocean upwelling plays a critical role in the abundance of NIT on surface waters of the western AS during JJA. Apart from NIT, the atmospheric soluble iron inputs are necessary for the growth of phytoplankton by consuming available N and P. Due to the shallow ocean mixing depths in the WAS, the upwelled waters have a low concentration of dissolved iron^14^. In this scenario, the Aeolian deposition is most likely the significant source of dissolved Fe^48^ in the WAS, where the abundance of DDF is high. The concentration of the dissolved iron decreases from east to west, whereas the phosphate and nitrate show an inverse pattern to the dissolved iron in the northeast AS^14^. In line with these, it is evident that WAS is rich in nitrates/dust due to upwelling/depositions making this region highly productive. The statistically significant correlation of NPP with NIT and DDF over the WAS (Fig. 4d) put further evidence showing the significant role of nitrates and dust depositions in the building-up of NPP over this region.

Unlike the WAS, dust deposition plays a significant role in NPP over the CAS. As seen in Fig. 4b, the spread of DDF is extended to the west coast of peninsular India. However, the upwelled nitrate spread (Fig. 4c) only up to 62°E longitude (5 µM L^−1^) under the influence of the prevailing northeast zonal ocean currents. Beyond that, towards the east, the concentration of nitrate falls below 1 µM L^−1^. The spread of NPP (Fig. 4a) also follows the same path as that of nitrates, but extending beyond 62°E even though the concentrations of nitrate are limited, but the dust deposition is still high. The limited spread of nitrate beyond 62°E and higher deposition of dust even beyond 62°E clearly highlights that dust deposition plays an important role in the building-up of NPP. However, the building-up of NPP could also be due to enhanced N_2_-fixation caused by the dust deposition. Guieu et al.^14^ have illustrated that if dust deposition is not taken into account, there is a drastic decrease in the N_2_-fixation over the AS. This was further supported by higher soluble iron concentrations in surface waters beyond 60°E leading to the higher N_2_ fixation rates. On the other hand, dust deposition on the ocean surface can upsurge the recycling of nutrients and carbon within the surface ocean^5^.

The correlation analysis has also shown a statistically significant association between NPP and DDF over the CAS than between NPP and NIT (Fig. 4e). However, the effect of various other ocean parameters on NPP cannot be ignored. Kumar et al*.*^19^ have illustrated the influence of ocean circulations in the building up of the chlorophyll on the CAS during JJA. It has been reported that the coastal upwelled waters in the west steadily move eastward, away from the west coast, under the influence of the prevailing easterly zonal currents. This leads to a strong downwelling in the CAS in coherence with the anticyclonic circulation in the sea. This downwelling of advected waters from the west deepens the ocean mixed layer depth (see Supplementary Fig. S4a online) over the CAS, limiting the supply of nutrients from deep ocean waters. Banerjee and Prasanna Kumar^32^ have identified a shallow mixed layer in the CAS region during winter monsoon using Price–Weller–Pinkel mixed layer model and illustrated that Chl-a over this region is prominently due to the dust deposition as the supply of nutrients is limited from the ocean. The CAS is also considered as the oligotrophic part of the open ocean. Jickells and Moore^5^ have documented that deposition of atmospheric dust is considered as an essential source of the micronutrient over the oligotrophic parts of the open ocean, where the inputs from other sources like river and groundwater discharge or sediment resuspension are minimal.

The inter-annual trends of DDF anomalies show statistically significant decreasing trends over the western and central AS. Similar to this, NPP also shows a decreasing trend, even though the trends are statistically not significant. The trend in anomaly is higher over the WAS (−5.8) as compared to that over central (−1.46) and eastern (−1.09) AS (Fig. 4f). As the decreasing trends in NPP correspond to the decreasing trends in DDF, it can be hypothesised that DDF has a considerable impact on NPP over the AS. Especially over the CAS, the association between NPP and DDF is statistically highest (r ~ 0.73), over which the trend in NIT is negligible (0.01).

In summary, the combined effect of nutrients from upwellings and dust depositions is crucial in building-up of NPP rates over western AS, whereas dust depositions alone play a significant role in building NPP over CAS where the availability of nutrients from ocean are limited. The linear associations and the decreasing trends indicate the suppression of NPP due to the reduction in mineral dust deposition over the AS on a long-term basis.

## Data and methodologies

### Dust detection, estimation of dust transport and deposition fluxes from CALIPSO

In this study, Cloud-Aerosol Lidar with Orthogonal Polarization (CALIOP) nighttime Level 2 version 4.20 aerosol profile (L2_05kmAPro) product^49^ is utilized. CALIOP is a two-wavelength (532 and 1064 nm) active satellite remote sensing instrument onboard CALIPSO^50^. The solar background illumination leads to a decrease in the signal-to-noise ratio during the daytime; hence CALIOP nighttime measurements have better quality than daytime^13,50^. To acquire only the high-quality data for the analysis, we applied the data screening scheme similar to Yu et al*.*^13^, with a strict cloud‐aerosol discrimination score between [−100, −90] to make our data cloud-free. The dust mass flux, deposition flux and loss frequency used in this study is derived following the steps (see Supplementary Fig. S5 online) described below. This technique is adopted from Yu et al*.*^13^.

The first step is to separate the pure dust from the mixed dust and generate the monthly mean three-dimensional dust extinction distribution. The method to separate pure dust is adopted from Amiridis et al*.*^51^ with a few modifications, where a characteristic dust lidar ratio of 44 sr was used instead of 58 sr, consistent with the number used in the CALIOP V4 product^52^. To partition the aerosol backscatter coefficient at 532 nm into dust and non-dust components, we used the most optimal separation method with the particulate depolarization ratio (PDR) thresholds for dust (0.2–0.3) and non-dust (0.07–0.02) particles, respectively^13,53^. Those values between dust and non-dust are considered the mixed dust PDR. For our current analysis, the mean values of the typical dust, non-dust, and mixed dust PDRs are used to estimate the dust backscattering coefficient. Dust extinction coefficient (DExt; Mm^−1^) is then obtained by multiplying the estimated dust backscattering coefficient by the characteristic dust lidar ratio of 44 sr. Second, to estimate the dust transport flux, we derived the dust mass concentration (DMC; units: g m^−3^) from the ratio of the so-obtained DExt and assumed dust mass extinction efficiency (DMEE). DMEE is the function of dust density and particle size, and it is estimated to be 0.37 m^2^ g^−1^ at 550 nm by Kaufman et al*.*^11^ based on several observations over the eastern tropical Atlantic ocean. Also, we assume that DMEE is independent of altitude and ambient relative humidity. The third step involves the estimation of zonal and meridional dust mass flux profiles (DMF; units: g m^−1^ s^−1^) by multiplying the obtained monthly three-dimensional dust mass concentrations, monthly mean wind vectors profiles (units: m s^−1^), and respective longitudinal (zonal)/latitudinal (meridional) lengths (units: m) of the segment. The monthly wind vectors profiles are obtained from the reanalysis of meteorological fields of the Modern‐Era Retrospective analysis for Research and Applications, Version 2 (MERRA2^54^). After estimating the profiles of zonal and meridional dust mass fluxes, the fourth step is to estimate the dust deposition flux using the flux divergence method^11,13^. It is assumed that there is no leak at the top of the atmosphere, and divergence is applied to column integrated zonal and meridional dust mass fluxes to derive the dust mass deposition flux (DDF; units: g s^−1^) into the AS. In the results, the units of flux are transformed to Tg year^−1^. Note that negative deposition fluxes are noticeable in this technique, and those values were neglected in the analysis since they are not physical^53^. The fifth step is to estimate the intensive property of dust called dust loss frequency (DLF; units: day^−1^). It is the ratio of the DDF (in g m^−2^ day^−1^) to the dust mass loading (units: g m^−2^) in the atmosphere^13,40^. The dust mass loading is the ratio of DOD to DMEE. DLF indicates how efficiently dust is removed from the atmosphere; hence larger the DLF, the more efficiently dust is deposited into the AS. In the previous step, it is seen that the DMF and deposition flux are dependent on the assumed DMEE, which leads to certain uncertainty. This DMEE related uncertainty can be cancelled out in estimating DLF by definition. Hence, better accuracy in understanding the dust loss in the atmosphere can be achieved than the estimated dust deposition flux. Also, the mass-weighted dust lifetime (DLT; units: days) in the atmosphere can be calculated by taking the inverse of DLF by definition^40^. Note that DLF measures how efficiently dust is removed, whereas DLT measures the timescale needed to remove dust in the atmosphere. To increase the representativeness of sampling, individual CALIOP profiles were regridded/aggregated into 2° × 2° horizontal resolution because of CALIOP's narrow cross‐track coverage. Note that all the other parameters used in this study have their respective fine spatial resolution. To have a smooth analysis for comparison between each parameter and to match each other, the spatial resolution was aggregated to a 2° × 2° grid. Whereas nitrate and ocean mixed layer depth retrieved from NOBM, the spatial resolution is aggregated to a 2° × 2.5° grid.

The accuracy of satellite-derived DDF can be tested using in-situ measurements. However, the study of DDF based on in-situ measurements over the AS is very limited. In order to understand the accuracy of DDF estimates in the present study, we have considered all the previous studies (estimated/simulated values) reported elsewhere over the north Indian Ocean (see Supplementary Table S1 online). As shown in Supplementary Table S1 online, the reported values of DDF over the north Indian Ocean show large variability from one observational/model simulation study to the other. It is observed that the reported values of DDF are higher than the annual value in the present study. However, our value of DDF is close enough to the reported value of Chester et al*.*^55^. Consideration of different dry and wet deposition schemes might cause significant variations in the reported values.

On the other hand, DMEE plays an important role in the accurate estimation of DDF from satellite-based observations. In the present study, DDF from CALISPO observations is estimated using the flux divergence method, including only the nighttime tracks. This might lead to underestimating the DDF values, as CALIPSO narrow cross-track leads to low spatial coverage. Further, it is to be noted that the geographic domain in the present study is only a part of the north Indian Ocean, while most of the values given in the Supplementary Table S1 online are for the entire north Indian Ocean. Apart from these, time scales are also different.

### Estimating the net primary productivity and nitrate concentration

In the present study, NPP is estimated using VGPM with inputs from MODIS. The monthly datasets of NPP were acquired with a spatial resolution of 0.16° × 0.16°. Since our analysis is on a decadal scale, monthly mean NPP data files are found reliable to understand the impact of dust on NPP. This is a "chlorophyll-based" model that estimates NPP from chlorophyll using a temperature-dependent description of chlorophyll-specific photosynthetic efficiency; hence it is a function of chlorophyll, available light, and the photosynthetic efficiency. An empirically parametrized SST-dependent polynomial fitting estimates the maximum daily net primary production of a given water column. There exist a few more models that provide the NPP, among them Eppley-VGPM (EVGPM)^56^ and Carbon-based Productivity Model (CbPM)^57^ models along with VGPM were used to validate NPP over the AS with measured values over the north AS (see Supplementary Fig. S6 online). The results are presented in Supplementary Table S2 online. It is seen that the VGPM model performed very well with the observed NPP values with the lowest RMSD (0.59) and bias (0.52) when compared with EVGPM and CbPM models.

The nitrate concentrations used in the present study are obtained from NOBM coupled with the Poseidon Ocean general circulation model with inputs from multiple satellite-derived and numerical models. This model provides four phytoplankton groups and four nutrient groups. The NIT used in the present study is one in the nutrient group. In addition, ocean mixed layer depth data is also used in this study from this model. This is an extensively used model and very well validated^58^. The NOBM data is available up to 2015 only. Hence, the mean spatial distribution and interannual variations of NIT and ocean mixed layer depth in the present study are calculated from 2007 to 2015. The comparison of mixed layer depth with in-situ observations over the Arabian Sea (see Supplementary Fig. S7 online) depicted very good spatial and inter-annual variability similar to that of the in-situ AGRO products considered for the period 2011–2015. The association between the two data sets is highest in the CAS (Pearson’s r = 0.93, RMSD ~ 15.68); whereas RMSD is lower in the WAS and EAS as compared to that in CAS. Similarly, the model simulated nitrate also showed very good association with the in-situ ship-based observation over the northern Arabian Sea (RMSD ~ 0.36 and correlation coefficient 0.77).

### Uncertainties in the satellite-based dust quantification

The satellite-based approach is subjected to uncertainties based on sensor type, viewing locations, instrument calibration errors, poor data accuracy, uncertainties in retrieval algorithms, and much more. In satellite-based quantification, active sources are more advantageous than any other passive instruments that cannot capture information like vertical profiles of aerosols and discrimination of aerosols and clouds^23^.

The above-estimated dust parameters can inevitably be subjected to uncertainties due to assumptions in implementing the adopted method. It is subjected to uncertainties associated with discrimination of pure dust from the mixed dust is 15–30%^12,51^, dust mass extinction efficiency^11^, assuming the dust transport height, possible change of dust size distribution during the transport and below-cloud dust^12^. The estimated dust transport flux from the temporal averages can also be subjected to the uncertainty of about 5%^11^ when dust concentration correlates strongly with wind fields. The estimation of dust deposition flux followed in this study has certain limitations. This approach is based on the mass balance between horizontal zonal and meridional dust mass fluxes in a respective grid cell; it is also assumed that there are no dust sources at the surfaces and no loss of dust from the top of the troposphere. Hence dust deposition fluxes are estimated only over the oceanic region. Moreover, this approach cannot distinguish wet and dry deposition; i.e., it gives the collective loss of dust from all removal mechanisms. The reliability of this dust deposition approach is validated by Yu et al*.*^13^ with 23 in-situ climatological dust deposition observations. It showed good agreement of about 65–83% but lower than the in-situ climatology by no more than 20%.

As discussed earlier, the assumed DMEE value is used to convert the DOD/extinction coefficient to the DMC; this DMC is utilised to estimate the DMF and, hence, DDF. The satellite-derived dust deposition is sensitive to the assumed DMEE value. In the present study, the DMEE value of 0.37 m^2^ g^−1^ reported by Kaufman et al*.*^11^ is used. However, DMEE can have large spatial heterogeneity. Hence, we carried out a comprehensive DMEE sensitivity analysis for annual dust deposition over the AS (see Supplementary Fig. S8 online). Based on the results obtained from Adebiyi et al*.*^59^ and Quinn et al*.*^60^, the mean DMEE over the Arabian Sea is 0.5 ± 0.2 m^2^ g^–1^. Quinn et al*.*^60^ calculated DMEE using Mie Calculations, and Adebiyi et al*.*^59^ obtained using Dust Constraints from joint Observational-Modelling-experiMental analysis (Dust-COMM) product. The sensitivity analysis of annual dust deposition for two different values of DMEE over the AS shows ~ 29% deviations for a change in DMEE from 0.37 m^2^ g^−1^ (used in the current analysis) to 0.5 m^2^ g^−1^. This difference (coefficient of variance is 19% for each of the cases of DMEE) is below the coefficient of variance (40%) reported by Quinn et al*.*^60^ over the AS.

In addition to DMEE, the selection of lidar ratio is another important parameter, which can also add considerable uncertainty to the satellite-based retrieval of dust concentration. Kim et al*.*^61^ have reported that the dust lidar ratio has large regional variability ranging from 30 to 60 sr. A sensitivity study of DOD over the Arabian Peninsula (AP) and the AS for different values of dust lidar ratio is shown in Supplementary Fig. S9 online. It is observed that the percentage difference in the DOD estimated from different values of lidar ratios varies from 33% in AP to 31% in AS, which is below the reported coefficient of variance of 61.6% over AP and 32.3% over the AS by Kim et al*.*^61^.

The errors in NPP obtained from VGPM model simulations are associated mainly with the chlorophyll concentration at the maximum C-fixation rate within a water column (P^b^_opt_). As satellite retrieved surface Chl concentrations are used in the model instead of depth-integrated Chl concentrations between euphotic depth and surface, this causes an estimated error in NPP of < 5%. The associated errors in the estimation of euphotic depth itself are < 15% while estimated using the case I model (as proposed by Morel and Berthon^62^), feeding surface Chl concentration as input. Behrenfeld and Falkowski^36^, give detailed uncertainties associated with NPP estimation using VGPM.

## Supplementary Information

Supplementary Information.

## Data Availability

All the datasets used in this study are open-source data: CALIPSO datasets are obtained from https://subset.larc.nasa.gov/calipso/login.php. The MERRA‐2 datasets are acquired from https://disc.gsfc.nasa.gov/datasets?project=MERRA-2. The Giovanni web portal: https://giovanni.gsfc.nasa.gov/giovanni/. Oregon State University Ocean Productivity website: http://sites.science.oregonstate.edu/ocean.productivity/index.php. NOBM datasets used in this study are available at https://gmao.gsfc.nasa.gov/gmaoftp/NOBM/monthly/.
