# Supplementary material for: On the net primary productivity over the Arabian Sea due to the reduction in mineral dust deposition
Source: Sci Rep. 2022 May 11;12:7761. doi: 10.1038/s41598-022-11231-7 (PMC9095634; doi:10.1038/s41598-022-11231-7)
Supplement: Supplementary file 1 — Supplementary Information. [file 41598_2022_11231_MOESM1_ESM.pdf]

On the net primary productivity over the Arabian Sea due to the reduction in mineral dust deposition

Chakradhar Rao Tandule[1], Mukunda M. Gogoi[2],
Rama Gopal Kotalo[1] and S. Suresh Babu[2]

[1]Aerosol & Atmospheric Research Laboratory, Department of Physics,
Sri Krishnadevaraya University, Anantapur 515003, India
[2]Space Physics Laboratory, Vikram Sarabhai Space Centre, ISRO,
Thiruvananthapuram, 695022, India

Supplementary Materials

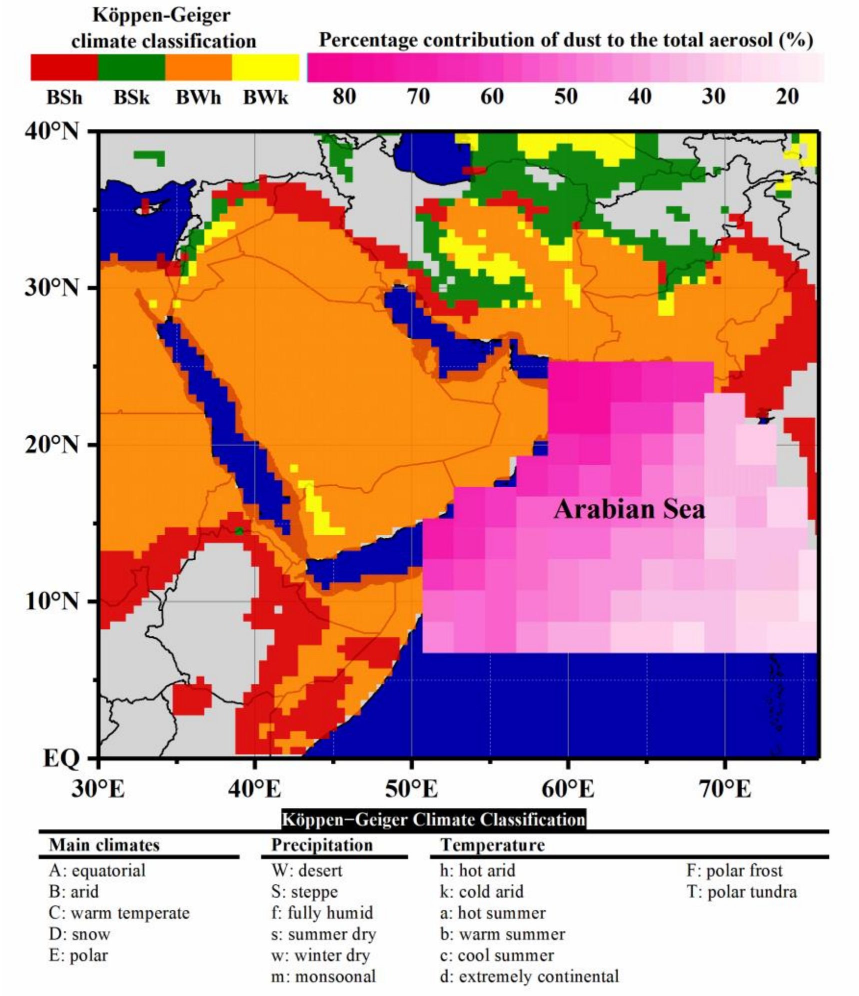

Figure S1: The map of the study domain, the Arabian Sea (8°N to 25°N, 51°E to 76°E) lite plum color contour representing the percentage contribution of dust to the total aerosol. The contour over land surfaces represents the Map of Köppen-Geiger climate classification; 'BSh' represents Arid, steppe, hot climate; 'BSk' represents Arid, steppe, cold climate; 'BWh' represents Arid, desert, hot climate; and 'BWk' represents Arid, desert, cold climate. The grey color represents the other climate regions/land surfaces, and the deep blue color represents the other ocean/major water-body regions.

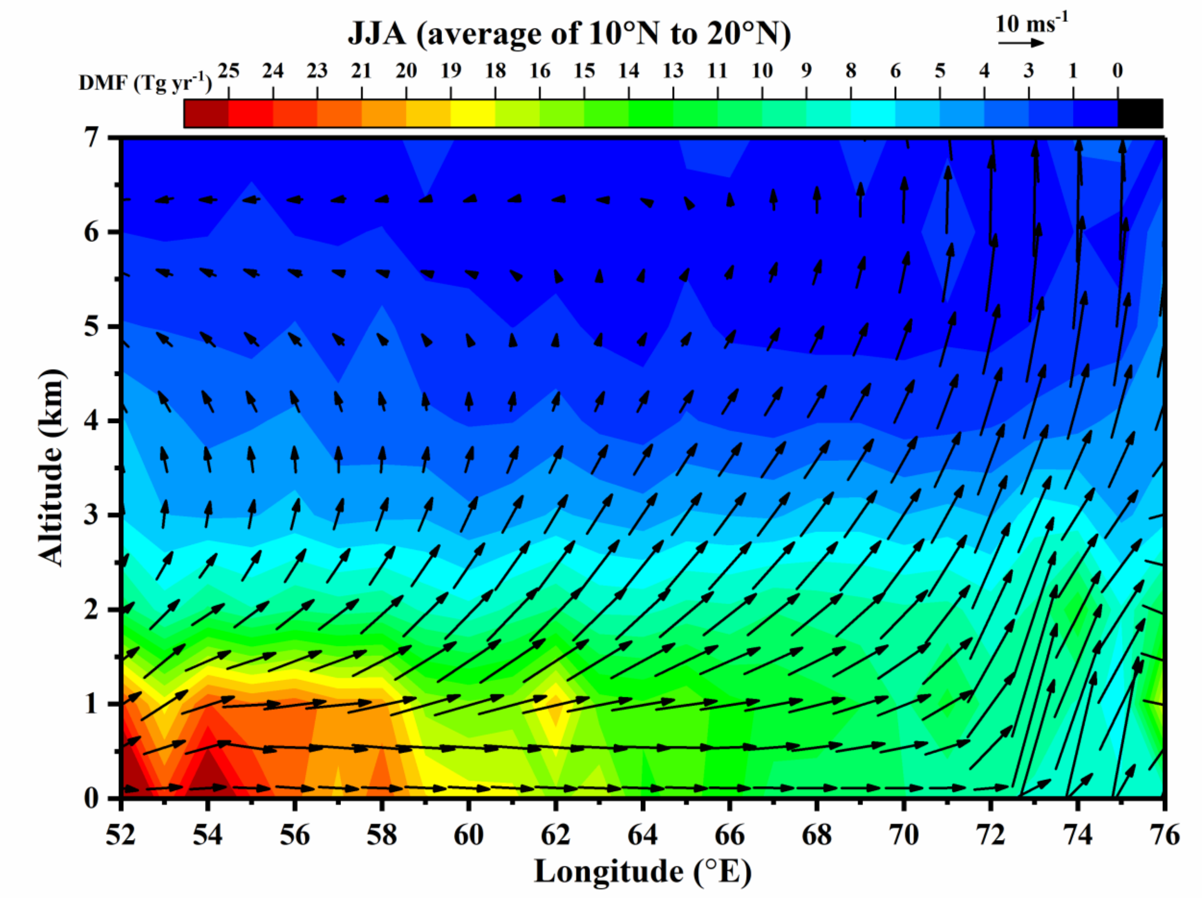

Figure S2: Longitude-altitude cross-section (average from 10°N to 20°N latitudes) of DMF over the AS during JJA (color contour). The arrow represents the vertical wind's direction, and the length represents the magnitude of the vertical wind.

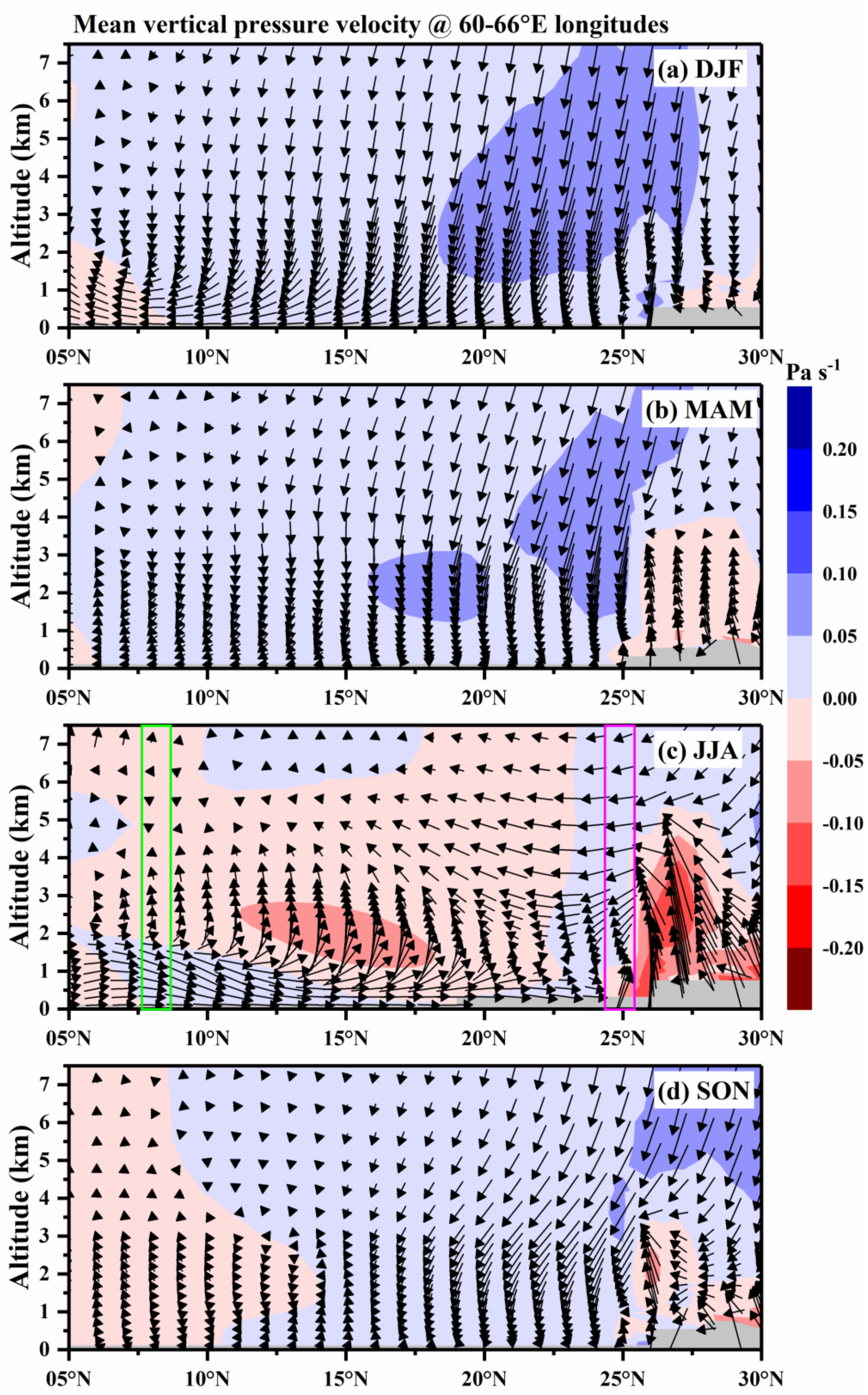

Figure S3: Latitude-altitude cross-section (60-66 °E longitude) of mean vertical pressure velocity at different seasons during (a) DJF, (b) MAM, (c) JJA, and (d) SON.

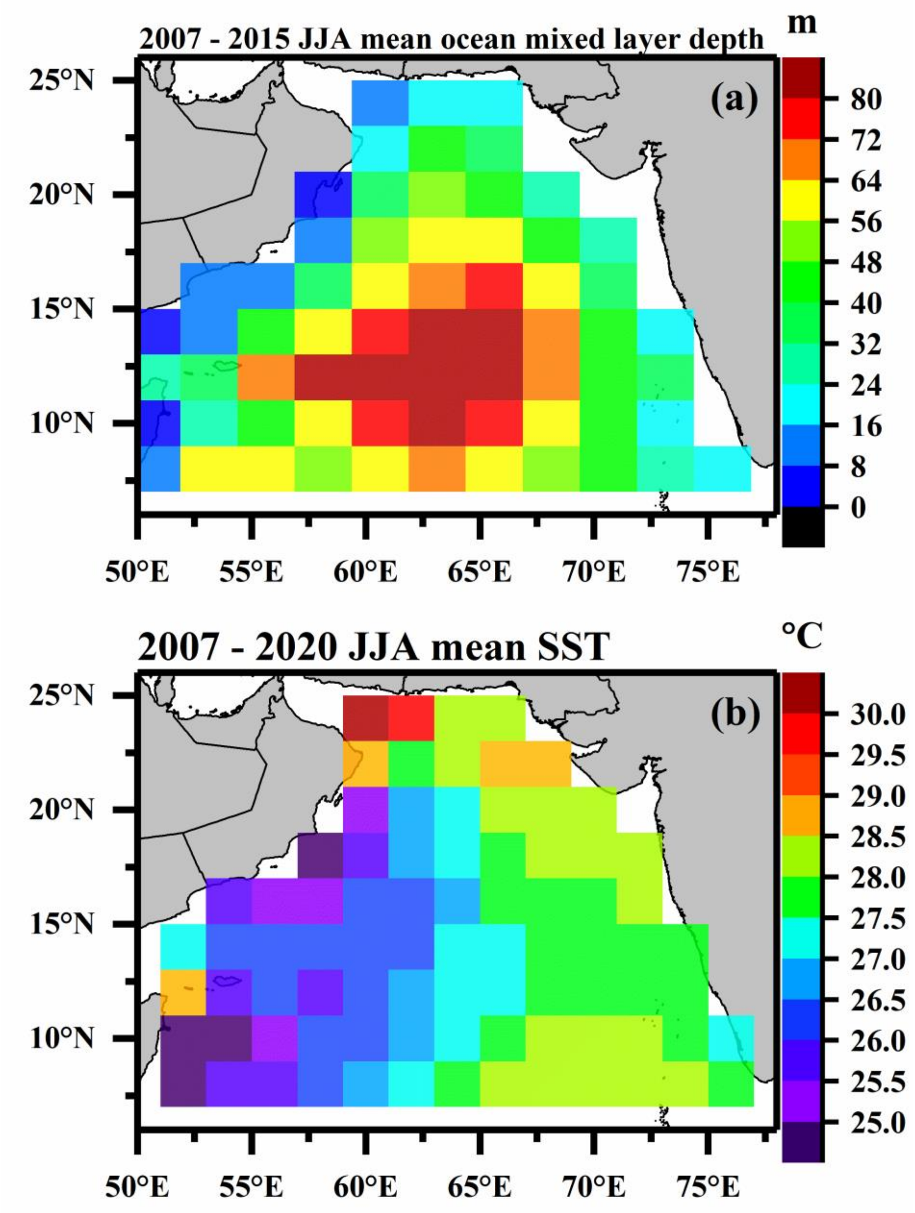

Figure S4: Spatial distribution of (a) the ocean mixed layer depth from 2007 to 2015 and (b) Sea surface temperature (SST) from 2007 to 2020.

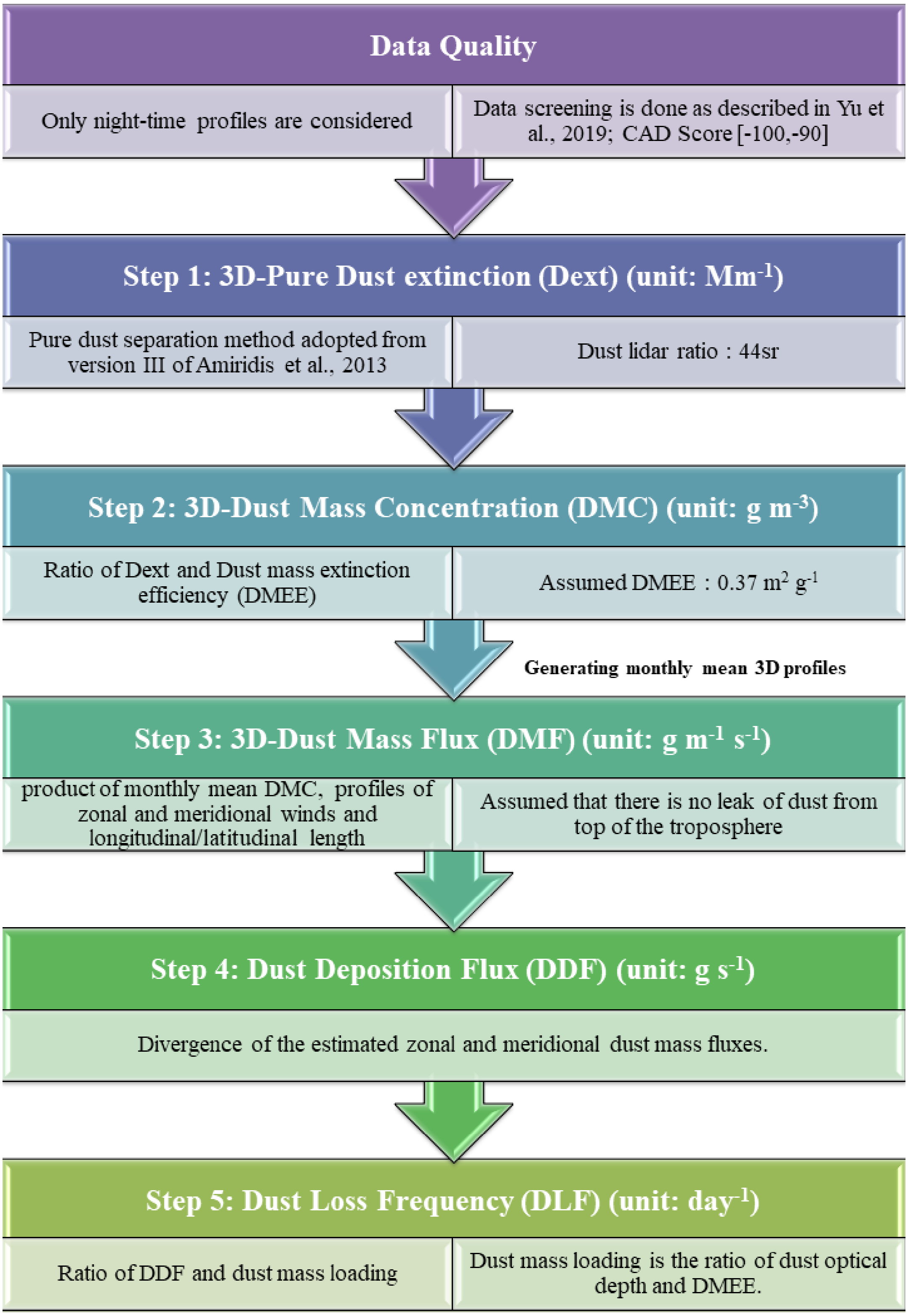

Figure S5: Flowchart of the methodology followed to calculate the dust mass flux, dust deposition flux, and dust loss frequencies over the study domain.

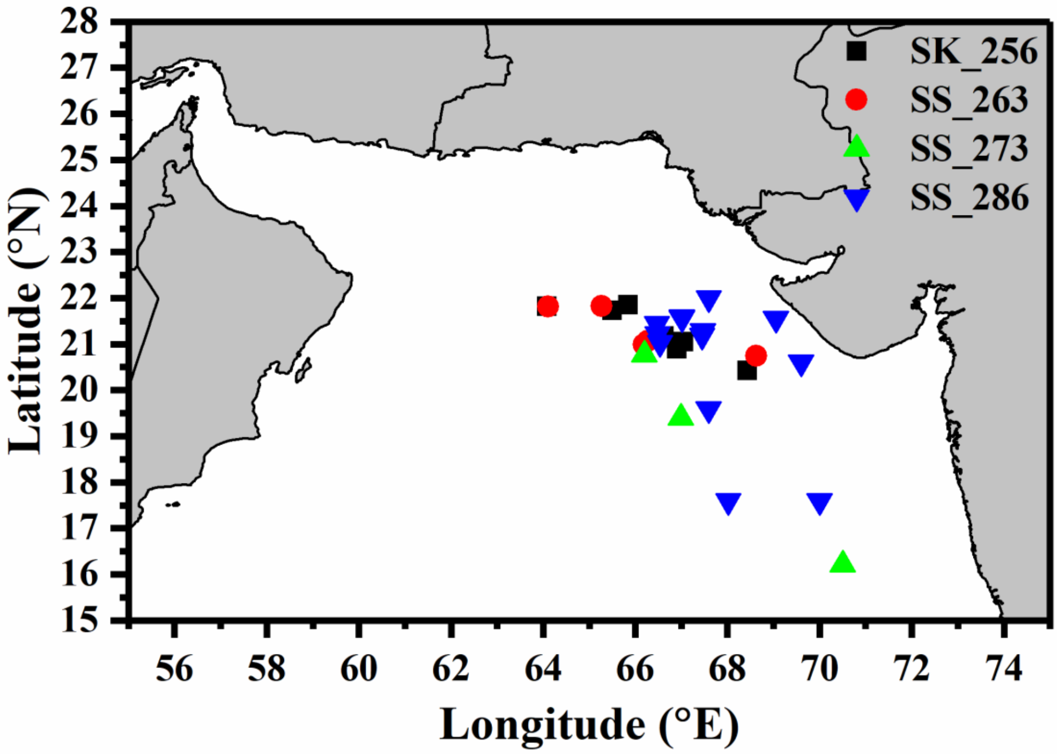

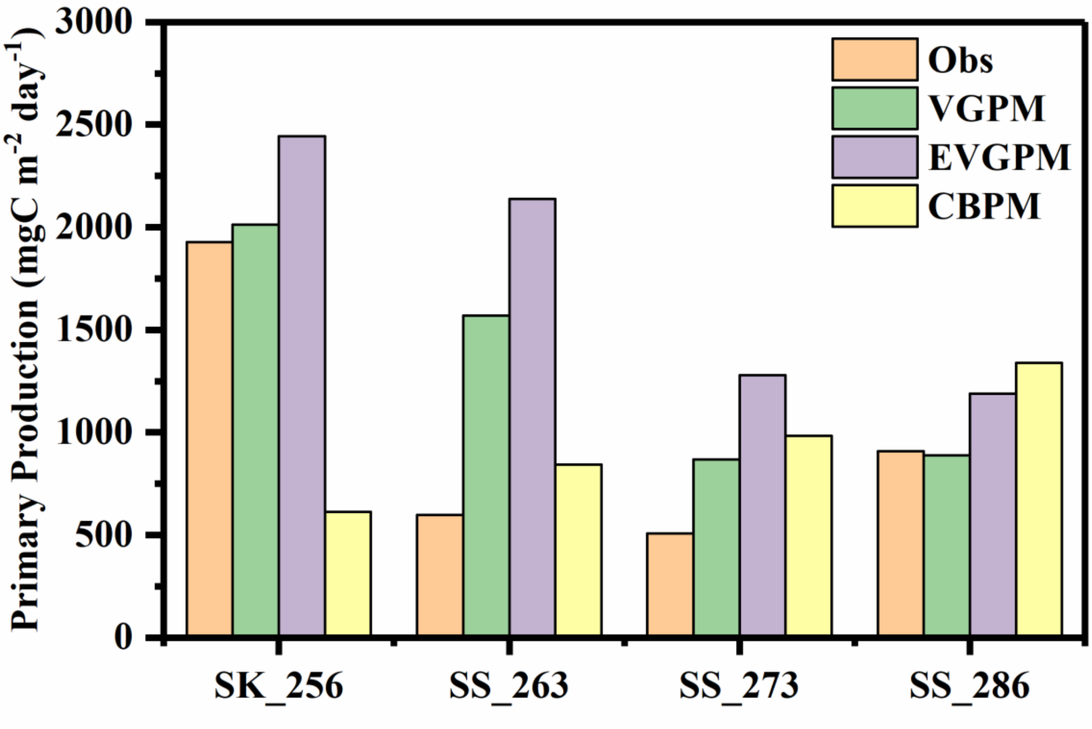

Figure S6: The locations of the in-situ samples used to estimate primary production in four cruises from 2009 to 2011 (top panel). Mean primary production values compared with those estimated from various models (bottom panel). The in-situ NPP data was acquired from R/V Sagar Kanya, R/V Sagar Sampada SK_256, SS_263, SS_273, SS_286 in the Northern Arabian Sea (Arabian Sea Noctiluca project)[1] for model validation.

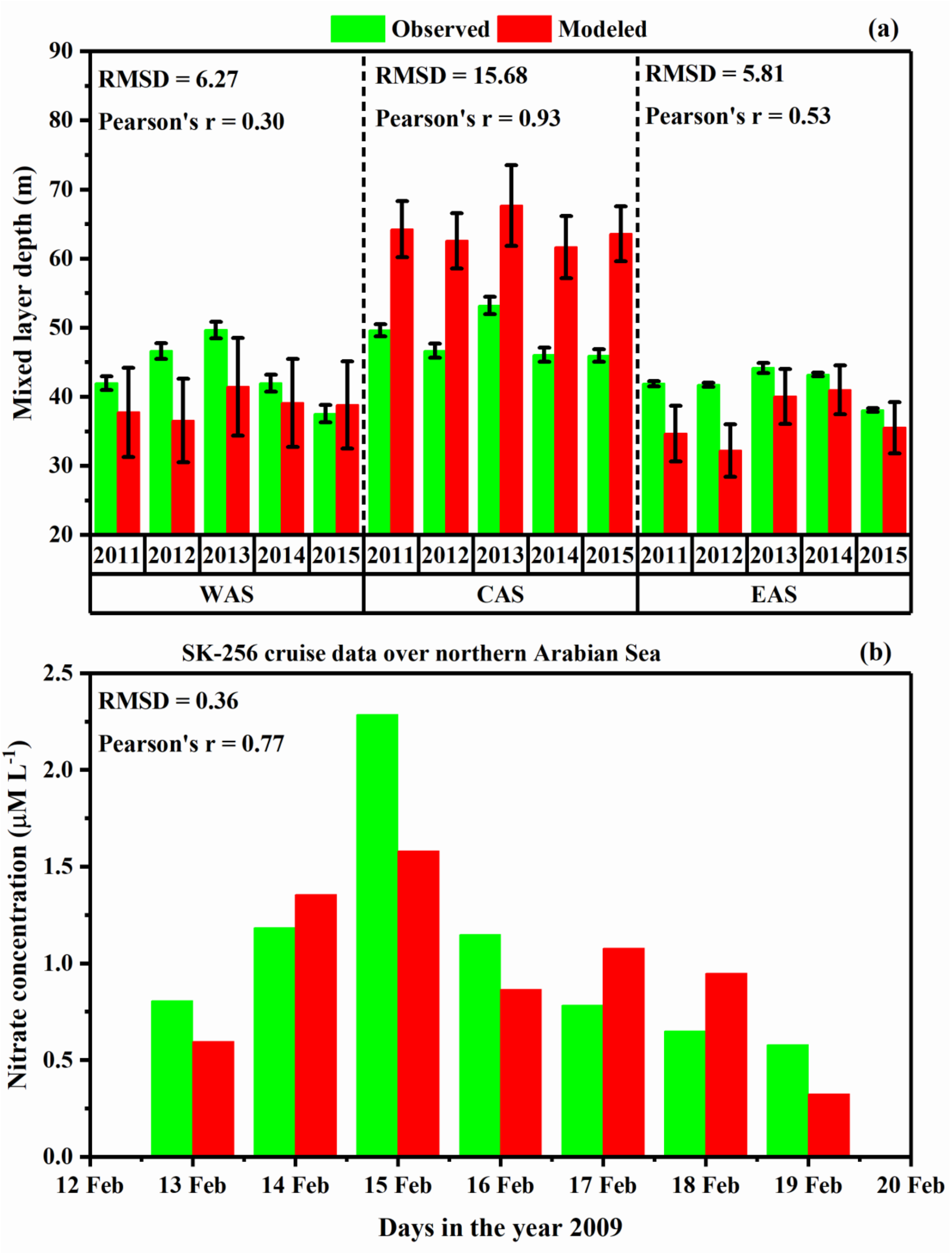

Figure S7: (a) Comparison observed and modelled regional mean mixed layer depth (1° x 1° interpolate Agro products, http://apdrc.soest.hawaii.edu/las/v6/dataset?catitem=9) for the years 2011-2015 over the Arabian Sea. Vertical bars indicating the standard error. (b) Daily mean nitrate concentrations from NOBM model, compared with in-situ observed data during 13 February to 19 February 2009 acquired from R/V Sagar Kanya, SK_256, in the Northern Arabian Sea (Arabian Sea Noctiluca project)1.

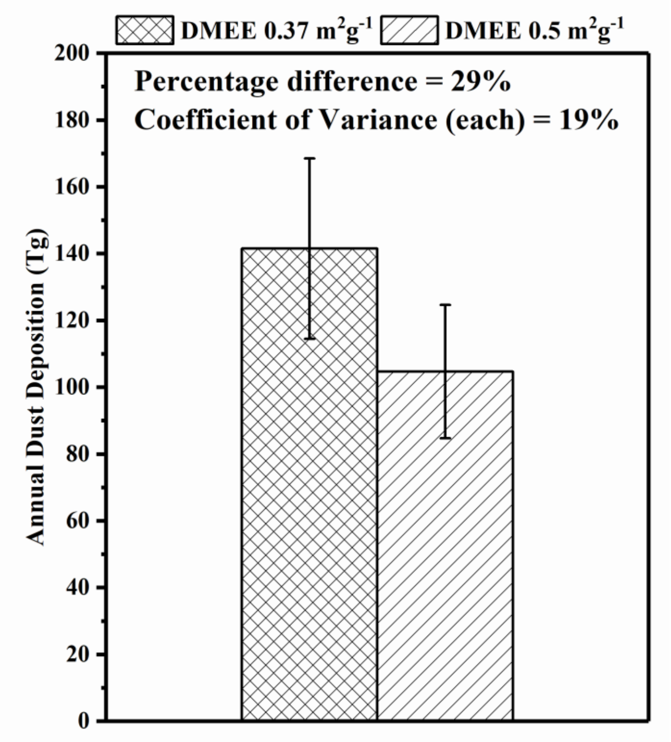

Figure S8: Sensitivity of 2007-2020 annual dust deposition over the Arabian Sea for the various assumed values of DMEE 0.37 m^2g^{-1} [2] and 0.5 m^2g^{-1}[3,4]. The values mentioned between the top of the bar is the percentage decrease with respect to 0.37 m^2g^{-1}.

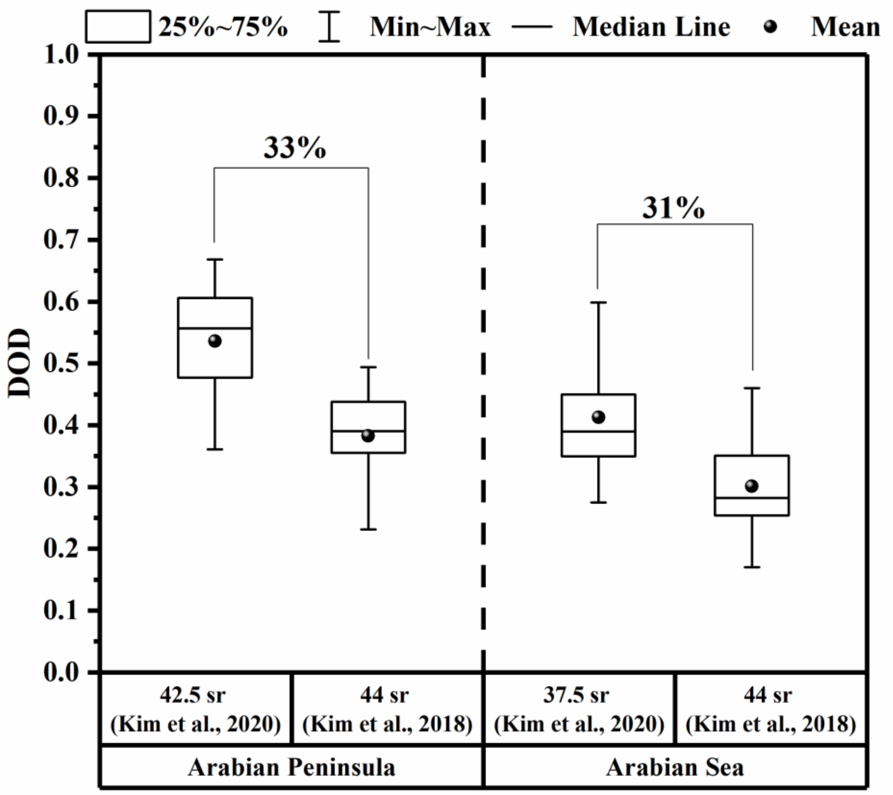

Figure S9: Sensitivity analysis for CALIPSO derived DOD over Arabian Peninsula (source region) and Arabian Sea (outflow region) with various lidar ratios. The value over each region is the percentage difference between mean DOD derived using two different lidar ratios over the respective region.

Table S1: Values of total annual dust deposition flux (Tg y^{-1}) over the northern Indian Ocean reported by earlier investigators.

DDF (Tg y^{-1})	Year of study	Reference	Remark
100	Annual 1975, 1979	*Duce et al.*[5]	Estimated using mean dry deposition velocity (0.2-0.4 cm s^{-1}) and scavenging ratio (1000). Dust concentrations were taken from *Prospero,*[6] and *Savoie et al.*[7].
20	Annual 1975, 1979	*Prospero,*[8]	It is modified from (*Duce et al.*[5]) using a scavenging ratio value of 200.
138	Annual 1987-1990, 1996	*Ginoux et al.*[9]	It was simulated using the GOCART model, where the scavenging parameter of sulfate is used to estimate dust deposition.
164	Annual 1981-1996	*Ginoux et al.*[10]	Simulated using the GOCART model
36	Annual 1990 - 1999	*Zender et al.*[11]	It is estimated using Mineral Dust Entrainment and Deposition (DEAD) model with a size-dependent washout coefficient.
1.6 - 28.6 (Tg)	Annual 1995	*Measures and Vink,*[12]	It is estimated using the excess Al seen in the mixed layer, assuming either 1.5 or 5% solubility of dust containing 8.2% Al by weight over the Arabia Sea.
10	October – November 1986	*Chester et al.*[13]	It is estimated using the measured Al and Al mineral ratio concentrations over the Northern Arabian Sea.
8.9	Annual (2007-2020)	Present study	It is estimated only over the Arabian Sea (northwest part of the Indian Ocean) using CALIPSO nighttime overpass).

Table S2: Statistical data of various NPP models compared with the in-situ data for the model validation purpose.

Model	RMSD	B
**VGPM (N = 07)**	**0.59**	**0.52**
**EVGPM (N = 07)**	**0.71**	**0.69**
**CBPM (N = 07)**	**0.83**	**0.81**

RMSD is the root mean square difference[14]
B is the bias[14]

References

1. Noctiluca project. Dataset: Phytoplankton and nutrients from R/V Sagar Kanya, R/V Sagar Sampada SK256, SS263, SS273, SS286 in the N. Arabian Sea from 2009-2011 (Arabian Sea Noctiluca project) | BCO-DMO. https://www.bco-dmo.org/dataset/3952.
2. Kaufman, Y. J. *et al.* Dust transport and deposition observed from the Terra-Moderate Resolution Imaging Spectroradiometer (MODIS) spacecraft over the Atlantic Ocean. *J. Geophys. Res. D Atmos.* **110**, 1–16 (2005).
3. Adebiyi, A. A. *et al.* Dust Constraints from joint Observational-Modelling-experiMental analysis (DustCOMM): Comparison with measurements and model simulations. *Atmos. Chem. Phys.* **20**, 829–863 (2020).
4. Quinn, P. K. *et al.* Aerosol optical properties during INDOEX 1999: Means, variability, and controlling factors. *J. Geophys. Res. Atmos.* **107**, 1–25 (2002).
5. Duce, R. A. *et al.* The atmospheric input of trace species to the world ocean. *Global Biogeochem. Cycles* **5**, 193–259 (1991).
6. Prospero, J. M. Mineral and Sea Salt Aerosol Concentrations in Various Ocean Regions. *J. Geophys. Res.* **84 C2**, 725–731 (1979).
7. Savoie, D. L., Prospero, J. M. & Nees, R. T. Nitrate, non-sea-salt sulfate, and mineral aerosol over the northwestern Indian Ocean. *J. Geophys. Res.* **92**, 933–942 (1987).
8. Prospero, J. M. *The Atmospheric Transport of Particles to the Ocean: Chapter 3 of 'Particle Flux in the Ocean: Scope 57'. JOHN WILEY & SONS Ltd., New York* vol. 57 (1996).
9. Ginoux, P. *et al.* Sources and distributions of dust aerosols simulated with the GOCART model. *J. Geophys. Res. Atmos.* **106**, 20255–20273 (2001).
10. Ginoux, P., Prospero, J. M., Torres, O. & Chin, M. Long-term simulation of global dust distribution with the GOCART model: Correlation with North Atlantic Oscillation. *Environ. Model. Softw.* **19**, 113–128 (2004).
11. Zender, C. S., Bian, H. & Newman, D. Mineral Dust Entrainment and Deposition (DEAD) model: Description and 1990s dust climatology. *J. Geophys. Res. Atmos.* **108**, 2013–2015 (2003).
12. Measures, C. I. & Vink, S. Seasonal variations in the distribution of Fe and Al in the surface waters of the Arabian Sea. *Deep. Res. Part II Top. Stud. Oceanogr.* **46**, 1597–1622 (1999).
13. Chester, R., Berry, A. S. & Murphy, K. J. T. The distributions of particulate atmospheric trace metals and mineral aerosols over the Indian Ocean. *Mar. Chem.* **34**, 261–290 (1991).
14. Regaudie-de-Gioux, A. *et al.* Multi-model remote sensing assessment of primary production in the subtropical gyres. *J. Mar. Syst.* **196**, 97–106 (2019).
